# Dynamics Analysis of Elastic Ring-Type Extruded Oil Film Damper Considering Time-Varying Characteristics

**DOI:** 10.3390/ma18091933

**Published:** 2025-04-24

**Authors:** Haibiao Zhang, Fuhua Liu, Tao Liu, Qingshan Wang

**Affiliations:** 1 College of Mechanical and Electrical Engineering, Central South University, Changsha 410083, China; 2 State Key Laboratory of Precision Manufacturing for Extreme Service Performance, Central South University, Changsha 410083, China; 3 Hunan Aviation Powerplant Research Institute, AECC, Zhuzhou 412002, China; 4 School of Civil Engineering, Central South University, Changsha 410075, China

**Keywords:** time-varying characteristics, elastic ring extruded oil film damper, two-way fluid-solid coupling, structural parameters, equivalent damping, equivalent stiffness

## Abstract

The elastic ring squeeze film damper (ERSFD), due to its compact structure and excellent mechanical properties, has been increasingly applied in various types of combination bearings for aero-engines. During operation, the force state of the elastic ring varies with different precession angles of the journal, leading to changes in the stiffness of the elastic ring. This study, based on a bidirectional fluid–structure interaction (FSI) theory, analyzes the deformation and stiffness of the elastic ring under different contact conditions. The time-varying stiffness curve of the elastic ring is obtained, and the influence of various parameters on its time-varying stiffness characteristics is further investigated. An equivalent stiffness method for the elastic ring is proposed, which improves accuracy by more than 3% at low speeds compared to traditional methods. Using this equivalent method, the effects of parameters such as the number of ring protrusions, protrusion width, protrusion angle, elastic ring thickness, and oil film eccentricity on the pressure distribution of the inner and outer oil films are analyzed. The results indicate that an increase in the number of elastic rings, protrusion width, axial length, and ring thickness leads to a rise in stiffness, with the number of protrusions having the strongest effect and the axial length having the weakest effect. Additionally, as the number of protrusions, protrusion width, and protrusion angle increase, both the damping and stiffness of the inner and outer oil films decrease by approximately 10%, with a more significant impact on the outer oil film than on the inner oil film. When the axial length and oil film eccentricity increase, both the damping and stiffness of the inner and outer oil films also increase, with the inner oil film being highly sensitive to eccentricity. However, excessive eccentricity enhances the nonlinearity of the oil film. The findings of this study provide a theoretical foundation for the design, application, and maintenance of combination bearings incorporating elastic ring squeeze film dampers.

## 1. Introduction

Modern turboshaft engines are high-speed rotating machinery, and their rotor is the most critical component, which directly affects the dynamic performance of the whole engine. The rotor in the aero-engine is mostly a combined rotor with an end tooth connection, which is mainly composed of the compressor and turbine disk, stepped shaft, and support system. In the process of rotor operation, due to the existence of factors such as the amount of unevenness and the existence of various order modes, there will always be unavoidable vibration. The vibration control of the rotor usually falls on the design of the support system. Conventional SFD systems are highly nonlinear and bistable due to static eccentricity and speed increase. In order to improve this phenomenon, Russian scholars designed an elastic ring and placed it in the oil film gap of the conventional SFD, forming the elastic ring squeeze film damper (ERSFD) [1]. The elastic ring divides the oil film into inner and outer parts, the inner and outer oil films are connected through small holes on the elastic ring, and the tabs on the elastic ring divide the inner and outer oil films into many small oil cavities. The inner tab of the elastic ring is generally in direct contact with the outer ring of the bearing, and the outer tab is in contact with the inner part of the elastic support [2]. In order to grasp the damping effect and dynamic characteristics of ERSFD, scholars at home and abroad have conducted a lot of research. Zhou Ming et al. established the control equations of ERSFD’s inner and outer oil film based on the simplified boundary conditions using Reynolds’ equation and calculated the stiffness and damping characteristics of the inner and outer oil films from them [3]. The damping effect and mechanism of ERSFD are studied and verified by experiments. It can be seen from the structure that not only is the oil film squeezed when the journal is fed but also the corresponding deformation is generated due to the better elastic–plastic effectiveness of the elastic ring. Based on the finite element theory, we calculated the effect of the deformation of the elastic ring on the force characteristics of the Hong Jie et al. ERSFD oil film by using the fluid–solid coupling method and also considered the effect of the ERSFD structural parameters on the characteristics of the oil film [4]. Cao Lei et al. investigated the effect of the stiffness characteristics of the elastic ring extruded oil film damper on the critical speed of the rotor system and experimentally determined that one of the important factors affecting the critical speed is the contact state of the elastic ring boss [5]. Su Chunfeng et al. established a finite element model of ERSFD considering the assembly tightness of the rigid ring and obtained the stiffness coefficients of ERSFD under different assembly tightnesses [6]. Zhang Wei et al. introduced the closed circular shell theory into the finite element model for studying the elastic ring, which better revealed the deformation of the elastic ring [7]. Xu et al. established the ERSFD finite element model considering the internal and external oil film connecting holes and explored the influence of the position of the connecting holes on the oil film force distribution [8]. Sun Kai et al. established a coupled ERSFD equation considering fit tightness to explore the impact of ERSFD fit tightness on the overall rotor dynamic characteristics. Furthermore, they proposed a matching design method for the structural parameters of the elastic ring using the particle swarm optimization algorithm [9,10]. Li Shengxiang et al. designed an ERSFD experimental system capable of precisely controlling static load application and investigated the fatigue life of the elastic ring through experiments [11]. Wang Shuhan et al. developed a dynamic analysis model coupling cylindrical roller bearings with ERSFD using a variable-step integration algorithm, providing recommendations on the matching of the roller cage and the elastic ring structural parameters [12]. Xiang Fengguang et al. introduced the dynamic parameters of the ERSFD into the rotor system and used the frequency sweep method to calculate the rotor’s dynamic characteristics, thereby improving the structural parameters of ERSFD [13]. Yang Minglin analyzed the force on the elastic ring structure, investigating the stress variations in the elastic ring under different structural parameters. He also analyzed the sensitivity of various parameters to the stress distribution, providing a reference for the fatigue life design of the elastic ring [14]. Chen Jie et al. used the finite difference method and finite element method to solve for the oil film pressure distribution, as well as the displacement and velocity of the elastic ring, respectively [15]. Xiaomin Yang developed a high-speed ball-bearing excited ERSFD model, with the deformation of the elastic ring analytically formulated. The effects of rotor operating speed and squirrel-cage stiffness on the transient and steady-state dynamics of the journal were examined [16]. Pang Guoying, according to the finite length bearing theory based on the partial differential equation, established four oil film pressure models and derived them to construct four contact models proposed by Russian scholars, considering the geometrical structure of the elastic ring with bosses and oil holes [17]. Zhong Luo established the mathematical model of the ERSFD–rotor system with coupling angular misalignment using the lumped mass method and analyzed the misalignment’s impact on the system. Additionally, the study explored the impact of the elastic ring’s structural parameters on the nonlinear vibration of the ERSFD–rotor system with coupling angular misalignment [18]. Wang Shuhan developed a dynamics analysis software for integrated cylindrical roller bearings with ERSFD and conducted simulation analyses on the oil film pressure and oil film forces after assembling the ERSFD onto the outer race of the bearing [19].

In summary, the main research on the elastic ring extruded oil film damper is centered on the deformation of the elastic ring, which is based on the computational analysis method of fluid–solid coupling to obtain the deformation of the elastic ring and then further obtain the related dynamic characteristics. However, the current study has not yet considered the dynamic deformation of the elastic ring due to the different contact states of the elastic ring, which lead to different stresses in the elastic ring. These will lead to changes in the stiffness of the elastic ring and the stiffness and damping of the inner and outer oil films, and these changes are time dependent. In view of this, this paper considers the force state of the elastic ring extruded oil film damper model, establishes the consideration of the time-varying stiffness and damping calculation method, and, on this basis, further analyzes and studies the elastic ring structural parameters caused by the change in the elastic ring extruded oil film damper dynamic characteristic change rule for the aero-engine combination of the supporting structure design to provide a theoretical method and engineering reference.

## 2. Modeling of an Elastic Ring Extruded Oil Film Damper

### 2.1. Definition of the Main Structure and Parameters of the ERSFD

**Figure 1:**
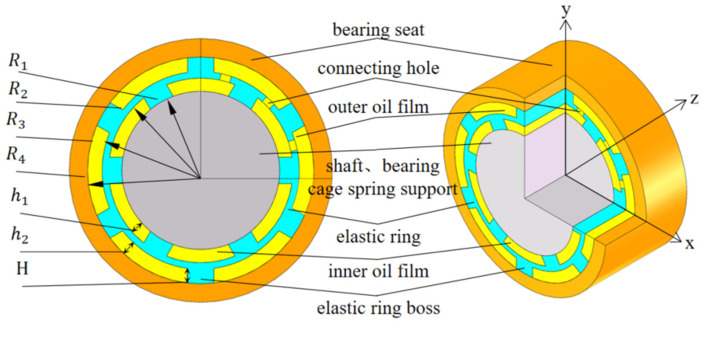
ERSFD structure and main parameters l.

The structure of the elastic ring-type extruded oil film damper (ERSFD) is shown in Figure 1, which consists of a shaft, a bearing, a squirrel-cage elastic support (which does not need to be considered separately in this study, so it has not been indicated), an inner oil film, an elastic ring, an outer oil film, and a support seat, respectively, from the inside out. The inner and outer walls of the elastic ring have a lot of tabs, which divide the inner and outer oil films into a lot of small oil cavities, and the elastic ring also has a connecting hole that connects the inner and outer oil films.

The expression for the thickness of the inner and outer oil films on the basis of not considering the deformation of the elastic ring, where the thickness of the inner oil film *h*_1_ is

(1) $$h_{1}=H$$

The thickness of the outer oil film $h_{2}$ is

(2) $$h_{2}=H$$

### 2.2. Oil Film Control Equation Without Considering the Deformation of the Elastic Ring

For the elastic ring-type extruded oil film damper, the oil film is divided into inner and outer oil films due to the existence of the elastic ring boss. Therefore, when establishing the oil film control equations, the different boundary conditions of the inner and outer oil films have to be considered separately to establish different fluid control equations.

**Figure 2:**
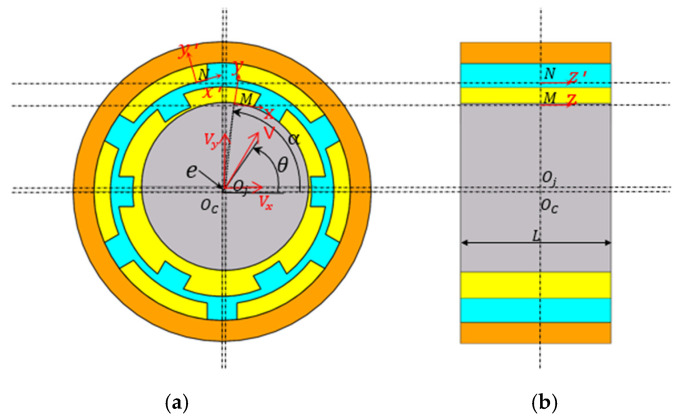
Eccentric coordinate system for ERSFD. (**a**) Front view of the ERSFD. (**b**) Side view of the ERSFD.

Due to unavoidable errors in design, machining, and assembly, the rotor–ERSFD system will always have a static eccentricity, as shown in Figure 2.

In Figure 2, *O_c_* is the center of the support seat, and *O_j_* is the center of the journal, which are coincident in the absence of static eccentricity. *e* is the eccentricity distance, θ is the eccentricity angle, *V* is the feed velocity, *x*, *y*, and *z* are the tangential, normal, and axial directions of the M-points, and *x*′, *y*′, and *z*′ are the tangential, normal, and axial directions of the N-points, respectively.

Combined with Equation (1), the thickness of the inner oil film can be derived at any point considering static eccentricity. Assume that the point is at an angle *α* in the horizontal direction:

(3) $$h_{1}=R_{2}-e\cos(\alpha -\theta )-\sqrt{e^{2}\cos(\alpha -\theta )-e^{2}+{R_{1}}^{2}}$$

Define the eccentricity ε=e/c, $c=R_{2}-R_{1}$ as the oil film gap and define λ=α−θ. Substituting into Equation (3) yields

(4) $$h_{1}=c(1-\varepsilon \cos\lambda );\frac{\partial h_{1}}{\partial \lambda }=-c\cdot \varepsilon \cos\lambda$$

According to the actual working boundary conditions of the inner and outer oil films, the inner and outer oil films’ control equations can be further obtained by combining the generalized Reynolds equation, without considering the deformation of the elastic ring.

Decompose the journal feed speed V into the speed in the direction of the lower x,y axis $V_{x},V_{y}$, and then determine the key parameters of the oil film boundary of the inner and outer oil films according to the corresponding boundary conditions.

For the inner oil film, the journal does inward movement and cannot be tampered with, and the elastic ring does not deform and cannot be axially tampered with. So, there are

(5) $$u_{0}=V_{x}\sin\alpha -V_{y}\cos\alpha$$

(6) $$v_{0}=V_{y}\sin\alpha -V_{x}\cos\alpha$$

(7) $$u_{h}=0,v_{h}=0$$

(8) $$w_{0}=0,w_{h}=0$$

Based on these boundary conditions, substituting Equations (5)–(8) into the Reynolds equation and combining them with the expression (1) for the thickness of the inner oil film $h_{1}$, the inner oil film control equation can be further expressed as

(9) $$\frac{\partial }{\partial x}\left(\frac{{h_{1}}^{3}}{\eta }\frac{\partial p}{\partial x}\right)+\frac{\partial }{\partial z}\left(\frac{{h_{1}}^{3}}{\eta }\frac{\partial p}{\partial z}\right)=6\frac{\partial }{\partial x}\cdot \left(\left(V_{x}\sin\alpha -V_{y}\cos\alpha \right)h_{1}\right)+12\left(V_{y}\sin\alpha -V_{x}\cos\alpha \right)$$

As for the outer oil film, the elastic ring does not deform and cannot be axially tampered with, and the support seat is regarded as a completely rigid body, so, there

(10) $$u_{0}=0$$

(11) $$v_{0}=0$$

(12) $$u_{h}=0,v_{h}=0$$

(13) $$w_{0}=0,w_{h}=0$$

Similarly, in conjunction with the expression for the outer oil film thickness $h_{2}$, the outer oil film control equation can be further expressed as

(14) $$\frac{\partial }{\partial x}\left(\frac{{h_{2}}^{3}}{\eta }\frac{\partial p}{\partial x}\right)+\frac{\partial }{\partial z}\left(\frac{{h_{2}}^{3}}{\eta }\frac{\partial p}{\partial z}\right)=0$$

From equation, it can be seen that there is no pressure on the outer oil film without considering the deformation of the elastic ring.

In order to conveniently obtain the pressure distribution in the circumferential direction of the oil film, the Reynolds equation for the inner oil film is directly written in column coordinate form by combining Equation (9) with

(15) $$\frac{\partial }{\partial \lambda }\left(\frac{h^{3}}{\eta }\frac{1}{r}\frac{\partial p}{\partial \lambda }\right)+\frac{\partial }{\partial r}\left(\frac{{h_{1}}^{3}}{\eta }\frac{\partial p}{\partial r}\right)=6r\Omega \frac{\partial h_{1}}{\partial \lambda }$$

Since the elastic ring extruded oil film damper, $L\ll B\frac{\partial p}{\partial r}\gg \frac{\partial }{\partial \lambda }$ can be assumed to be an infinitely short bearing to find the approximate solution. Equation (15) can be simplified as

(16) $$\frac{\partial }{\partial r}\left(\frac{{h_{1}}^{3}}{\eta }\frac{\partial p}{\partial r}\right)=6r\Omega \frac{\partial h_{1}}{\partial \lambda }$$

Integrating Equation (5) into Equation (17) yields

(17) $$\frac{\partial }{\partial r}\left(\frac{r{h_{1}}^{3}}{\eta }\frac{\partial p}{\partial r}\right)=-6r\Omega c\cdot \sin\lambda$$

Using the semi-Sommerfeld boundary conditions, the condition that the oil film cannot withstand negative pressure in the dispersion zone is considered, and assuming that the pressures in the dispersion zone are all zero, i.e., the boundary conditions are formulated as

(18) $$\begin{array}{l} p(\lambda ){|}_{\lambda =0}=p(\lambda ){|}_{\lambda =\pi \sim 2\pi }\\ p(\lambda ,r){|}_{r=\frac{R_{2}-R_{1}}{2}}=0 \end{array}$$

Integrating Equation (18) twice into the boundary conditions gives an approximate solution to the ERSFD short-bearing Reynolds equation as

(19) $$p(\lambda ,r)=\frac{6r\eta \Omega c_{\varepsilon }\sin\lambda }{{h_{1}}^{3}}\left(\frac{{\left(R_{2}-R_{1}\right)}^{2}}{4}-r^{2}\right)$$

Integrate the solved oil film pressure along the axial r and circumferential λ directions, respectively, and substitute Equations (1)–(6) to obtain the radial force $F_{r}$ and the tangential force $F_{t}$, respectively.

(20) $$\left\{\begin{array}{c} F_{r}=\frac{\eta \Omega \varepsilon {\left(R_{2}-R_{1}\right)}^{3}}{c^{3}}\left[\frac{2\varepsilon^{3}}{{\left(1-\varepsilon^{3}\right)}^{2}}\right]\\ F_{t}=\frac{\eta \Omega \varepsilon {\left(R_{2}-R_{1}\right)}^{3}}{c^{3}}\left[\frac{\pi \varepsilon }{2{\left(1-\varepsilon^{2}\right)}^{\frac{3}{2}}}\right] \end{array}\right.$$

The equivalent oil film stiffness $K_{o\_in}$ and equivalent oil film damping $C_{o\_in}$ of the oil film inside the ERSFD extruded oil film damper can be further obtained from the tangential and radial forces.

(21) $$\left\{\begin{array}{c} K_{o\_in}=-\frac{F_{r}}{e}=\frac{\eta \Omega \varepsilon {\left(R_{2}-R_{1}\right)}^{3}}{c^{3}}\left[\frac{2\varepsilon }{{\left(1-\varepsilon^{3}\right)}^{2}}\right]\\ C_{o\_in}=-\frac{F_{t}}{e\Omega }=\frac{\eta \varepsilon {\left(R_{2}-R_{1}\right)}^{3}}{c^{3}}\left[\frac{\pi }{2{\left(1-\varepsilon^{2}\right)}^{\frac{3}{2}}}\right] \end{array}\right.$$

### 2.3. Analysis of Elastic Ring Deformation

In the actual working conditions, the calculation of the oil film gap should not only take into account the eccentricity of the oil film itself because the elastic ring, due to low stiffness, will be deformed to a certain extent when it is subjected to the load of the force of the journal inlet and the pressure of the oil film, which will also lead to a change in the thickness of the oil film.

**Figure 3:**
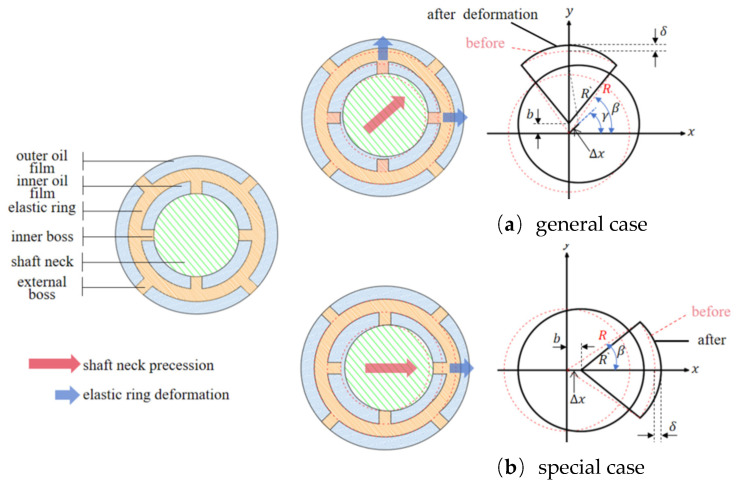
Different deformation scenarios of the elastic ring: (**a**) general case; (**b**) special case.

The direction of journal movement is different, so the deformation of the elastic ring is different at different moments. This paper, according to the number of stressed cams, will be divided into two cases, as shown in Figure 3.

In Figure 3, δ is the deformation amount of the elastic ring, γ is the angle of advancement, and Δx is the amount of advancement. Figure 3a is the general case. In this case, the main contact force points of the journal and the inner ring of the elastic ring tab are two or more, and the outer rings of the elastic ring and the inner ring of the support seat contact tab force points are three or more, and the deformation of the elastic ring can be decomposed into the neighboring elastic ring shaft segments on each of the two tabs being considered in order to facilitate the discussion later on; this case is called the double-tab force case.

Figure 3b is a special case, which occurs only when the journal’s feed angle is within the angle range of the inner cam. In this case, the stress point of the inner cam of the elastic ring only exists in the corresponding cam position, while the stress point of the outer cam only exists in two because the deformation of the elastic ring and the direction of the feed are the same in this case, and, each time, the singular number of the inner cam is directly stressed, so this case is called the single cam stress case.

**Figure 4:**
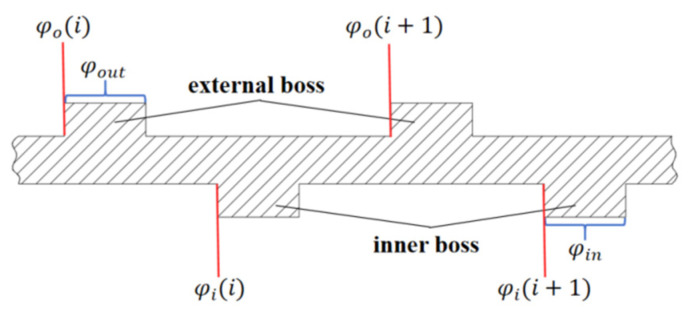
Corresponding angles of the inner and external bosses.

Based on these two different deformation cases, the deformation of the elastomer ring at the position corresponding to the angle θ can be obtained, and since the thickness of the elastomer ring is very thin, it can be assumed that the deformation of the elastomerized inner and outer rings at the same position is equal. Assuming that there are n outer tabs and there are n inner tabs, each of which has width D, the corresponding angles of the outer tabs are $\varphi_{out}$, the corresponding angles of the inner tabs are $\varphi_{in}$, the starting angle of each inner tab is set to be $\varphi_{0}\left(i\right)$ (i = 1, 2, 3, …, n), and the starting angle of the inner tabs is set to be $\varphi_{i}\left(i\right)$ (i = 1, 2, 3, …, n), as shown in Figure 4.

According to Figure 4, the corresponding feed angles for the two different cases can be obtained, and in the double-bump stress case, the range of the feed angle γ at this time is

(22) $$\left\{\begin{array}{cc} 1<i<n-1 & \varphi_{i}(i)+\varphi_{in}<\gamma <\varphi_{i}(i+1)\\ i=n & \varphi_{i}(n)+\varphi_{in}<\gamma <\varphi_{i}(1) \end{array}\right.$$

You can obtain the positional deformation δ at the angle θ as

(23) $$\delta =\left\{\begin{array}{cc} 0 & \left|\theta -\gamma \right|\geq 90^{\circ }\;\mathrm{or}\varphi_{o}(i)<\theta <\varphi_{o}(i)+\varphi_{\mathrm{out}}\\ \Delta x\cos(\theta -\gamma ) & \theta =\frac{\varphi_{o}(i)+\varphi_{o}(i)+\varphi_{o}(i+1)}{2}\;\mathrm{and}\;\left|\theta -\gamma \right|<90^{\circ }\\ \Delta x\cos(\theta -\gamma )\cdot \cos\left(\left|\theta -\varphi_{in}+\frac{\varphi_{in}}{2}\right|\right) & \varphi_{o}(i)+\varphi_{\mathrm{out}}<\theta <\varphi_{o}(i+1)\mathrm{and}\;\left|\theta -\gamma \right|<90^{\circ } \end{array}\right.$$

In the case of a single-bump force condition, the range of the feed angle γ is, at this time,

(24) $$\varphi_{i}(i)<\gamma <\varphi_{i}(i)+\varphi_{in}$$

At this time, the journal deformation along the corresponding position of the cam is symmetrical, and the deformation δ is

(25) $$\delta =\left\{\begin{array}{cc} 0 & \left|\theta -\gamma \right|\geq 90^{\circ }\mathrm{or}\varphi_{o}(i)<\theta <\varphi_{o}(i)+\varphi_{\mathrm{out}}\\ \Delta x & \theta =\frac{\varphi_{o}(i)+\varphi_{o}(i)+\varphi_{o}(i+1)}{2}\mathrm{and}\left|\theta -\gamma \right|<90^{\circ }\\ \Delta x\cos\left(\left|\theta -\varphi_{in}+\frac{\varphi_{in}}{2}\right|\right) & \varphi_{o}(i)+\varphi_{\mathrm{out}}<\theta <\varphi_{o}(i+1)\mathrm{and}\left|\theta -\gamma \right|<90^{\circ } \end{array}\right.$$

**Figure 5:**
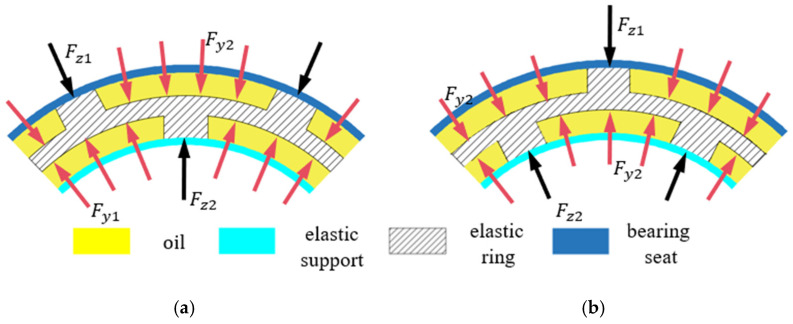
Force diagram of the elastic ring. (**a**) Single-bump force case. (**b**) Double-bump force case.

In the circumferential direction, since the rotation of the elastic ring is restricted, i.e., the friction between the inner and outer cams can be neglected, the elastic ring is mainly subjected to four kinds of axial loads, namely, the load force of the inner journal (the outer ring of the squirrel-cage spring support) on the inner cam $F_{z2}$, the support force of the inner ring of the support seat on the outer cam $F_{z1}$, the pressure of the inner film on the inner ring of the elastic ring $F_{y1}$, and the pressure of the outer film on the outer ring of the elastic ring, and the force diagrams are as follows. With the oil film pressure on the outer ring of the elastic ring $F_{y2}$, the force diagram is in Figure 5.

The overall thickness of the elastic ring is very thin, and the height of the cam is much smaller than the thickness of the elastic ring, so the vibration of the elastic ring can be approximated by using the thin-plate deformation theory.

Based on the force of the elastic ring, the transverse vibration differential equation of the elastic ring can be established:

(26) $$K\nabla^{4}\delta +m\frac{\partial^{2}\delta }{\partial t^{2}}=F_{z2}+F_{z1}+F_{y1}+F_{y2}$$

Combined with the previous analysis of the deformation case of the elastic ring, the expression of the elastic ring stiffness $K_{ersfd},$ considering the working case of the elastic ring, can be further obtained:

(27) $$K_{ersfd}=\frac{0.129SEn^{4}s^{3}L_{z}}{\left(R_{m}-0.3b_{e}n^{3}\right)}\cdot \frac{1}{\left[1-\left(1-\frac{S^{3}}{{H_{m}}^{3}}\right)\left(1.45A-0.9A^{2}+0.2A^{3}\right)\right]}$$

In Equation (27), $A=\frac{bn}{R_{m}}$, $R_{m}=\frac{R_{1}{+R}_{4}}{2}H_{m}=R_{4}{-R}_{2}$, and $S=L_{z}b_{e}$. $b_{e}$ is the equivalent width of the elastic ring, for example, if only one tab is in full contact, $b_{e}=b$, and if two tabs are in full contact, $b_{e}=2b$. So only S is time varying, and the others are constants, so the constant part is equal to Λ. Therefore, we can further obtain the expression for the time-varying stiffness of the elastic ring $K_{ersfd}$ (t):

(28) $$K_{ersfd}\left(t\right)=\frac{S(t)}{\left[1-\left(1-\frac{S{\left(t\right)}^{3}}{{H_{m}}^{3}}\right)\right]}\cdot \Lambda$$

### 2.4. Oil Film Control Equation Considering Elastic Ring Deformation

After obtaining the movement and deformation of the elastic ring, according to the actual case of the elastic ring work, the expression for the thickness of the inner and outer oil films can be derived on the basis of considering the deformation of the elastic ring, where the thickness of the inner oil film $h_{1}$ is

(29) $$h_{1}=c(1-\varepsilon \cos\lambda )+\delta$$

(30) $$\frac{\partial h_{1}}{\partial \lambda }=-c\cdot \varepsilon \cos\lambda +\frac{\partial \delta }{\partial \lambda }$$

The thickness of the outer oil film $h_{2}$ is

(31) $$h_{2}=H-\delta$$

(32) $$\frac{\partial h_{2}}{\partial \lambda }=\frac{\partial \delta }{\partial \lambda }$$

For the inner oil film, due to the effect of the deformation of the elastic ring,

(33) $$v_{h}=\frac{\partial \delta }{\partial t}$$

Similarly, the stiffness and damping expressions for the inner and outer oil films can be obtained.

For the inner oil film, the stiffness $K_{o\_in}$ and damping $C_{o\_in}$ are

(34) $$\left\{\begin{array}{c} K_{o\_in}=\frac{H^{3}}{{\left(H+\delta \right)}^{3}}\frac{\eta \Omega \varepsilon {\left(R_{2}-R_{1}\right)}^{3}}{c^{3}}\left[\frac{2\varepsilon }{{\left(1-\varepsilon^{3}\right)}^{2}}\right]\\ C_{o\_in}=\frac{H^{3}}{{\left(H+\delta \right)}^{3}}\frac{\eta \varepsilon {\left(R_{2}-R_{1}\right)}^{3}}{c^{3}}\left[\frac{\pi }{2{\left(1-\varepsilon^{2}\right)}^{\frac{3}{2}}}\right] \end{array}\right.$$

The stiffness $K_{o\_out}$ and damping $C_{o\_out}$ of the outer oil film are

(35) $$\left\{\begin{array}{c} K_{o\_in}=\frac{\partial \delta }{\partial t}\frac{H^{3}}{{\left(H-\delta \right)}^{3}}\frac{\eta \Omega \varepsilon {\left(R_{2}-R_{1}\right)}^{3}}{c^{3}}\left[\frac{2\varepsilon }{{\left(1-\varepsilon^{3}\right)}^{2}}\right]\\ C_{o\_in}=\frac{\partial \delta }{\partial t}\frac{H^{3}}{{\left(H-\delta \right)}^{3}}\frac{\eta \varepsilon {\left(R_{2}-R_{1}\right)}^{3}}{c^{3}}\left[\frac{\pi }{2{\left(1-\varepsilon^{2}\right)}^{\frac{3}{2}}}\right] \end{array}\right.$$

## 3. ERSFD Fluid–Structure Coupling Analysis and Time-Varying Stiffness Analysis of Elastic Ring

### 3.1. Introduction to ERSFD Bidirectional Fluid–Solid Coupling

**Figure 6:**
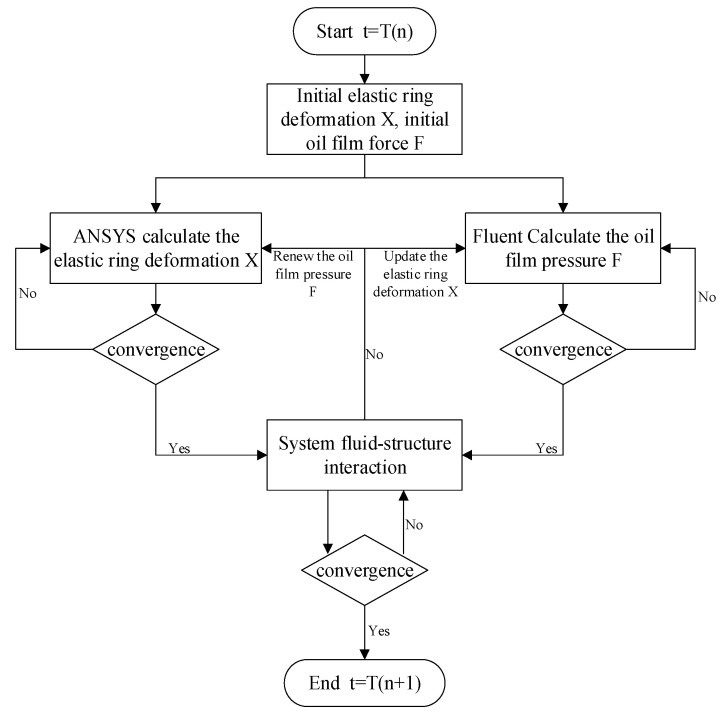
Bidirectional fluid–structure interaction.

In this paper, ANSYS 2022R1 Workbench finite element software is used to analyze the two-way fluid–solid coupling of ERSFD oil film structure, in which the transient structural dynamics of the elastic ring, the squirrel-cage elastic support, the support seat, and other structures are analyzed in the solid part, and the transient hydrodynamics analysis of the internal and external oil film and other fluid parts are analyzed in the Fluent module. For the sake of data uniformity, the two parts share the same geometry and the same pre-processing data. The analysis process of two-way fluid–solid coupling is shown in Figure 6.

Assuming that at the moment T(n), the elastic ring and the inner and outer oil films have an initial deformation X and oil film pressure F, which is 0 at the initial moment, then it is mainly the external excitation or the X and F converged at the previous moment. At this time, the initial inner and outer oil films’ motion and the elastic ring force and deformation are substituted into the corresponding solvers and iterated, respectively. When both the solid structure solver and the fluid structure solver converge, the internal and external oil film pressure F calculated by the fluid structure and the elastic ring deformation X calculated by the solid structure are substituted into the two-way fluid–solid coupling solver, and the mesh interpolation and mutual exchange are used to determine whether the coupling converges or not. If the coupling does not converge, the internal and external oil film force F obtained from the fluid structure calculation is used as the initial condition for the solid structure calculation, and the elastic ring deformation X obtained from the solid structure calculation is used as the initial condition for the fluid structure calculation to carry out iterative calculations again until the fluid–solid coupling system converges, and when the fluid–solid coupling system converges, the oil film force and the elastic ring deformation of the moment are outputted as the results of the calculations and as the initial condition for the next moment, and the oil film force and the elastic ring deformation of the next moment are outputted as the results of the calculations. When the fluid–solid coupling system converges, the oil film force and the elastic ring deformation at this moment are outputted as the calculation result and as the initial condition for the next moment, and the next moment is calculated.

### 3.2. Analysis of Solid-Coupled Deformation in Elastic Circulation

**Figure 7:**
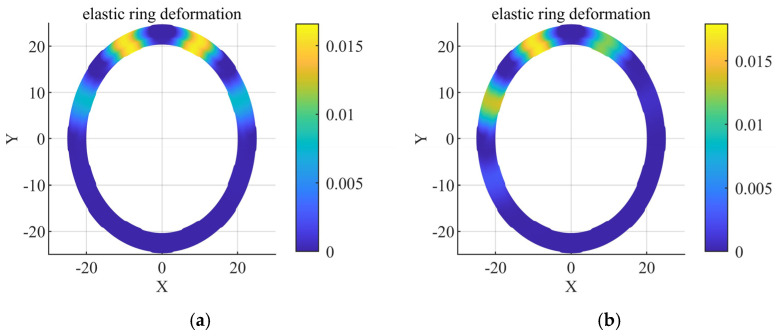
Radial deformation comparison. (**a**) Double-bump stresses. (**b**) Force on a single tab.

In order to study the effect of the action of internal and external oil film fluid–solid coupling on the deformation of the elastic ring, a load with variable direction is applied to the journal. The results are compared with the action without considering the internal and external oil film fluid–solid coupling, and the deformation of the elastic ring is analyzed by taking two states, respectively, in which Figure 7a is the double-bump force case, and Figure 7b is the single-bump force case.

Through the calculation as well as the ANSYS simulation, it is understood that the deformation of the whole axial elastic ring is consistent, so the deformation of the elastic ring can be analyzed by obtaining any one of the middle cross-sections, and the deformation data of the symmetric plane are obtained here for discussion. According to the data in Figure 7a,b, the deformations on the inner walls of the elastic rings in the two states at the maximum deformation position are 0.0156 mm and 0.0169 m, while the deformations on the outer walls are 0.0162 mm and 0.0174 mm, respectively, which are less than 3%, indicating that the deformations of the inner and outer walls at the same circumferential position are nearly the same. This is due to the fact that when the elastic ring is subjected to force, the direction of force is always perpendicular to the inner and outer surfaces of the elastic ring, so when the deformation is not very large, the elastic ring will not be deformed in other directions. This result is also consistent with the assumption of the elastic ring as a thin-walled structure in our previous theoretical analysis and verifies the correctness of the previous theoretical analysis.

So, the deformation of the elastic ring would only need to be explored along the circumferential direction, and the analysis can be further simplified to one on a random cross-section of the elastic ring. Here, a circle with the same distance from the inner and outer tabs of the elastic ring is taken, and the deformation of ERSFD is probed around the deformation on this circle.

**Figure 8:**
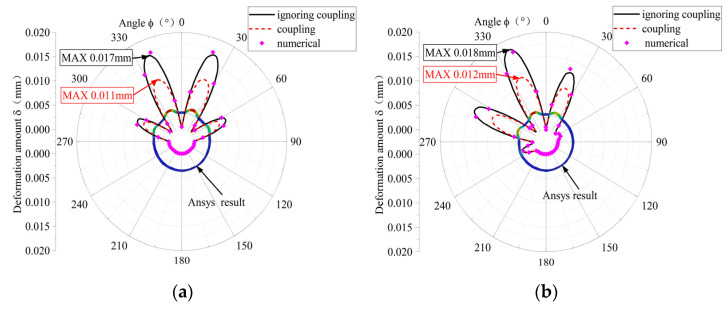
Circumferential deformation distribution. (**a**) Double-bump stresses. (**b**) Force on a single tab.

The circumferential deformation of the elastic ring, obtained by considering the fluid–structure coupling and not considering the fluid–structure coupling, and the numerical calculation for the two cases are shown in Figure 8, respectively. The results of the numerical calculations in the figure are basically consistent with the simulation results, which verifies the correctness and reliability of the model. From Figure 8a,b, it can be seen that the deformations of the elastic rings without considering the fluid–solid coupling are 0.017 mm and 0.018 mm, while the deformations of the elastic rings with the consideration of the fluid–solid coupling are 0.011 mm and 0.012 mm, respectively, which are about 35% lower than that of the deformations without considering the fluid–solid coupling on the average, because after considering the fluid–solid coupling, the inner and outer oil films will form a dynamic pressure with the inward movement of the elastic ring. The reason is that after considering the fluid–solid coupling, with the movement of the elastic ring, the inner and outer oil films will form a dynamic pressure oil film, which generates a certain amount of oil film pressure on the corresponding shaft segments to reduce the deformation of the elastic ring, and the more obvious the deformation of the elastic ring, the greater the oil film pressure.

**Figure 9:**
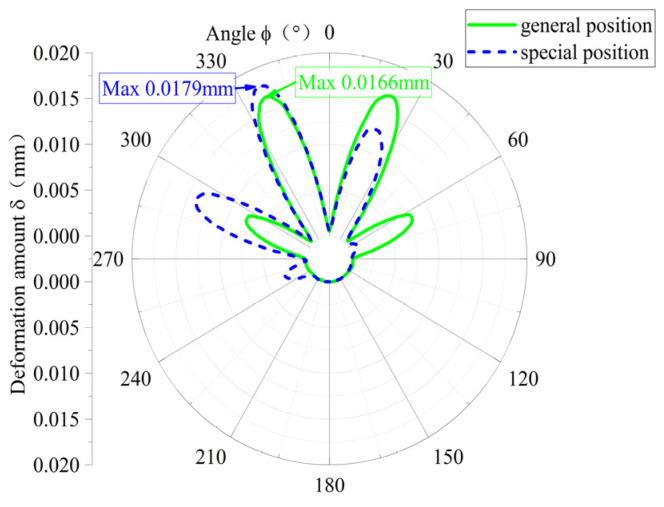
Comparison of elastic ring deformation.

In Figure 9, the circumferential distribution of the deformation of the elastic ring is compared between the single-bump stress case and the double-bump stress case when the loads are consistent. The deformation of the elastic ring in the single-bump stress case is concentrated, with a large deformation in only three adjacent tab segments, and the deformation distribution is basically symmetric, with a maximum deformation of about 0.0180 mm, while the deformation of the elastic ring in the double-bump stress case is more dispersed, with a large deformation in more than four tab segments, but the maximum deformation is only 0.0166 mm, with the maximum deformation of both cases being close to 10% different. The difference between the two maximum deformations is close to 10%.

**Figure 10:**
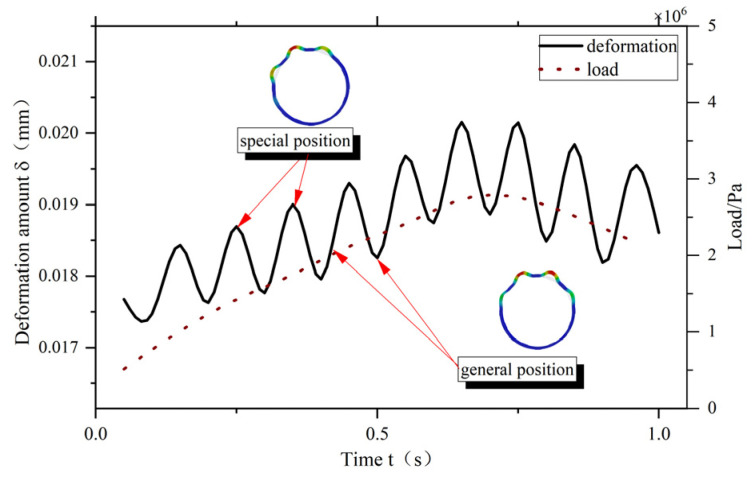
Variation in maximum deformation of the elastic ring with load change.

In order to further investigate the deformation of the elastic ring at different positions, Figure 10 shows the maximum deformation of the elastic ring at different moments when the load is varied at a certain frequency. When the load changes, the maximum deformation of the elastic ring is not linearly followed by the increase but appears similar to the trigonometric function of the periodicity, and the period is about 0.125 s. The maximum deformation is always larger and then smaller to show a periodic shape. Because the load applied here not only changes in size with time but also changes in direction with time, when the load direction changes, the elastic ring is always converted between the single-bump force state and the double-bump force state, which leads to a change in the maximum deformation of the elastic ring. The corresponding moments of each extreme value are the moments of the single-bump force state of the elastic ring, and the other moments are the double-bump force state.

### 3.3. Analysis of the Effect of Different Structural Parameters on the Time-Varying Stiffness of the Elastic Ring

During finite element analysis, the stiffness K of the elastic ring can be defined as

(36) $$K=\frac{1}{n}\sum_{i=1}^{n}\frac{F_{i}}{\delta_{i}}$$

**Figure 11:**
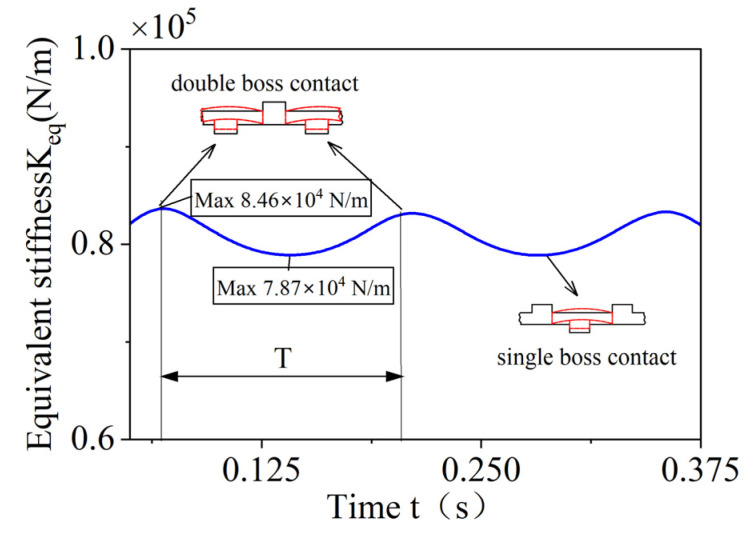
Time-dependent stiffness curve of the elastic ring.

In Equation (36), *n* is the number of nodes, and $F_{i}$ and $\delta_{i}$ are the load and displacement of the corresponding nodes, respectively. According to Equation (35), combined with the deformation of the elastic ring and the applied load, the time-varying stiffness curves of the elastic ring can be obtained by considering the oil film fluid–structure coupling, as shown in Figure 11.

The time-varying stiffness period of the elastic ring is the time that the load passes through two adjacent single-bump stress positions, and when the elastic ring is in the single-bump stress position, the elastic ring stiffness is the lowest, which is $7.87\times 10^{4}\;N/m$, and when the inlet position is at the corresponding angle of the outer cam, the elastic ring stiffness is the largest, which is $8.46\times 10^{4}\;N/m$, and the stiffness difference between the two positions is about 10%.

In order to further investigate the time-varying stiffness characteristics of the elastic ring, it is found that the elastic ring parameters, such as the number of tabs, the width and height of the tabs, the axial length of the ring, and the thickness of the elastic ring, have an effect on the stiffness of the elastic ring by combining with Equation (26). Further, different parameters of the elastic ring are varied to perform two-way fluid–solid coupling simulations to investigate the variation in the corresponding time-varying stiffness characteristics of the elastic ring.

**Figure 12:**
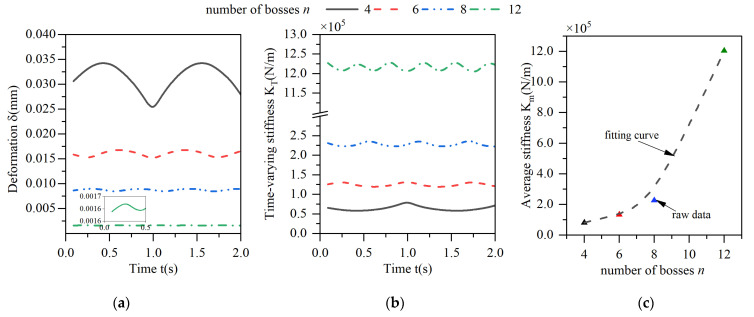
Effect of changes in the number of bosses on the deformation and stiffness of the elastic ring. (**a**) Deformation of the elastic ring. (**b**) Time-varying stiffness of the elastic ring. (**c**) Mean stiffness change.

The effect of the variation in the number of tabs on the peripheral stiffness characteristics is shown in Figure 12, where graph (a) shows the deformation of the elastic ring, graph (b) shows the time-varying stiffness of the elastic ring, and graph (c) shows the average stiffness of the elastic ring during one cycle of the time-varying stiffness curve.

As the number of tabs increases, the average deformation of the elastic ring decreases, and the stiffness increases. When the number of tabs increases from four to twelve, the deformation of the elastic ring decreases by about 90%, and the average stiffness increases by a factor of nearly 10. This is because when the number of tabs of the elastic ring increases, the span of the beam corresponding to its deformation decreases, and although the number of chambers of the oil film increases, the overall area of the oil film decreases, which leads to an increase in the overall stiffness. As the number of tabs increases, the period of time-varying stiffness decreases, and the time-varying characteristics are weakened. The change in the stiffness of the elastic ring becomes smaller and smaller, with a change in stiffness of about 25% at four tabs and less than 10% at twelve tabs.

The period T of the time-varying stiffness can be further calculated from Figure 12 as a function of the number of tabs N as

(37) $$T=\frac{T_{F}}{N}$$

where $T_{F}$ is the frequency of the load, and the period is exactly the time from one positive advance position to another.

**Figure 13:**
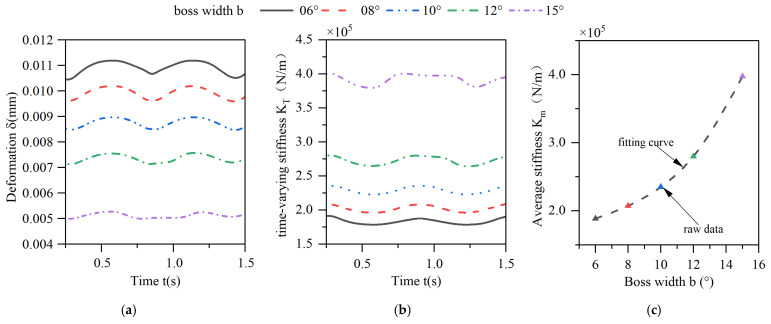
Effect of changes in the boss width on the deformation and stiffness of the elastic ring. (**a**) Deformation of the elastic ring. (**b**) Time-varying stiffness of the elastic ring. (**c**) Mean stiffness change.

The characteristics of the deformation of the elastic ring and its time-varying stiffness by the change in the width of the elastic ring tabs are shown in Figure 13. As the width of the tabs increases, the deformation of the elastic ring decreases, and the time-varying stiffness of the elastic ring increases gradually, and the elastic ring with a tab width of 15° has an approximately 200% increase in the time-varying stiffness compared to that of the elastic ring with a tab width of 6°. Consistent with the principle of the effect of increasing the number of tabs as the width of the tabs increases, the span of the beam corresponding to the deformed position decreases, corresponding to a decrease in the width of the oil film, thus increasing the stiffness. From Figure 13b, it can be seen that the time-varying stiffness property of the elastic ring is weakened when the width of the tabs is increased, and its curve has a straight line at the cusp position, which is due to the fact that the increase in the width of the tabs leads to an increase in the region of the elastic ring in the state of the single-bump force, and it appears that the stiffness does not change for a short period of time, and this property becomes more and more obvious with the increase in the width of the tabs.

**Figure 14:**
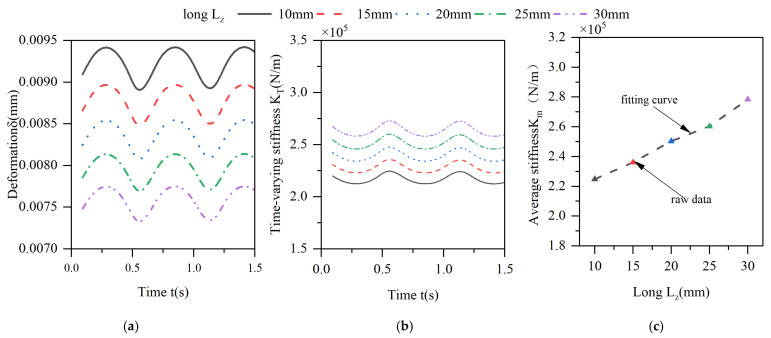
Effect of changes in the length of the elastic ring on its deformation and stiffness. (**a**) Deformation of the elastic ring. (**b**) Time-varying stiffness of the elastic ring. (**c**) Mean stiffness change.

The effect of the axial length of the elastic ring on the deformation of the elastic ring is shown in Figure 14. With the increase in the axial length, the deformation of the elastic ring also shows a decreasing trend, but the change is not very obvious. When the thickness is increased from 10 mm to 30 mm, the amount of change in the elastic ring decreases by only about 20%. The effect of the same increase in tab width on the time-varying stiffness characteristics of the elastic ring is only reflected in the fact that the overall stiffness shows an increasing trend. This is due to the fact that the increase in the width of the elastomerization does not change the span of the deformed beam of the elastic ring, nor does it change the thickness of the oil film; it only increases the area under force and thus increases the stiffness. Moreover, as the area under force increases, the reaction force provided by the oil film pressure increases, which also further enhances the stiffness of the elasticized ring.

**Figure 15:**
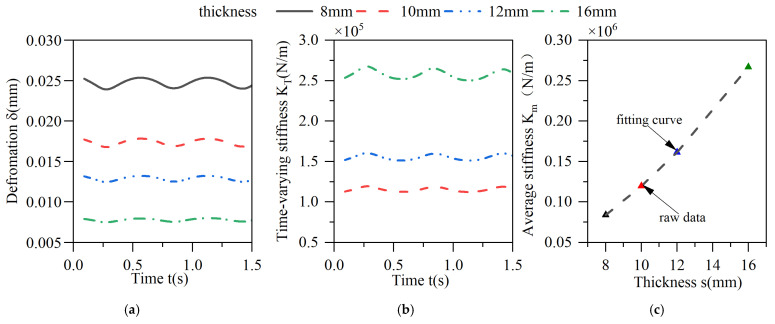
Effect of changes in the thickness of the elastic ring on its deformation and stiffness. (**a**) Deformation of the elastic ring. (**b**) Time-varying stiffness of the elastic ring. (**c**) Mean stiffness change.

The effect of the thickness of the elastic ring on the deformation of the elastic ring and its stiffness is shown in Figure 15, where the inner and outer diameters of the elastic ring remain unchanged and only the thickness changes.

As the thickness of the elasticized ring decreases, although the thickness of the corresponding oil film increases, the reduction in the wall thickness of the elasticized ring directly reduces the cross-sectional area of the corresponding beam, resulting in a very significant change in stiffness, and when the wall thickness is reduced from 16 mm to 8 mm, the deformation of the entire elasticized ring increases by 150%, and the average stiffness is reduced by 75%. As the thickness of the oil film increases with the reduction in the elastomerization thickness, the time-varying stiffness characteristics of the elastic ring become more and more sensitive, and the amount of change in the stiffness of the elastic ring becomes larger and larger, and the amount of change in the stiffness is about 10% when the thickness of the elastic ring is 16 mm, while the amount of change in the stiffness is more than 20% when the thickness of the elastic ring is 8 mm.

The degree of influence of the various parameters of the elastic ring on the maximum deformation and average stiffness of the elastic ring is given in Table 1. All the degrees of variation are standardized for the elastic ring with eight tabs, a width of 10°, a thickness of 10 mm, and a length of 15 mm, and the variation in various parameters at the standard parameters is zero.

In order to obtain a more intuitive picture of the influence of each parameter of the elastic ring on the deformation and equivalent stiffness of the elastic ring, a parameter for the degree of influence is defined ϵ:

(38) $$\in =\frac{\left|P\right|}{\left|X_{i}-X_{0}\right|}\cdot X_{0}$$

**Figure 16:**
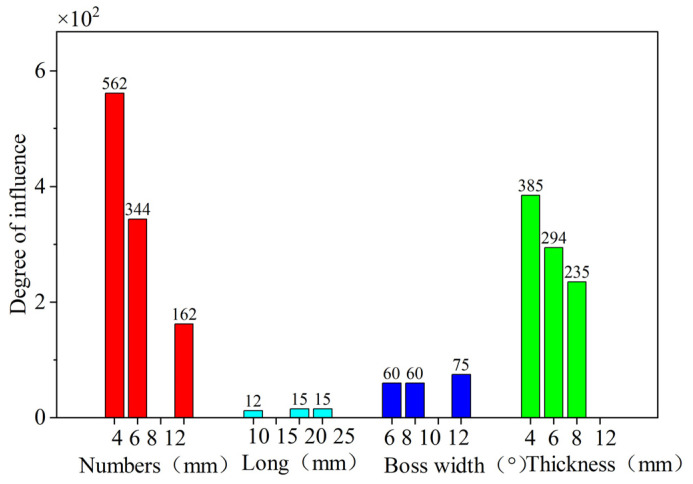
Influence of various parameters on the deformation and stiffness of the elastic ring.

Figure 16 compares the degree of influence of various elastic ring parameters on the equivalent stiffness of the elastic ring, from which it can be learned that the number of elastic ring tabs, elastic ring tab width, and elastic ring thickness have a very large influence on the stiffness of the elastic ring, while the axial length of the elastic ring has relatively less influence, and the number of tabs and the elastic ring thickness have a very significant influence on the time-varying stiffness characteristics of the elastic ring.

Therefore, the subsequent design of the elastic ring parameters should give priority to the first three parameters for consideration and, when it is necessary, to consider the time-varying stiffness characteristics of the elastic ring but also the number of tabs and the thickness of the elastic ring for further investigation.

### 3.4. Establishment of Equivalent Stiffness Method for Elastic Ring Considering Time-Varying Characteristics

The elastic ring has been simplified to a thin-plate structure in the previous section, and due to the uniformity of the force distribution of the elastic ring in the axial direction, the elastic ring can be further simplified to be considered a simple beam. So, the equivalence is carried out by using a consistent strain energy increment over a period of time, and assuming that the stiffness does not change without a change in load, the strain energy increase after a period of time t is

(39) $$U\cdot t=\int_{0}^{\delta_{1}}Pd\delta \cdot t=\int_{0}^{\delta_{1}}K_{eq}d\delta \cdot t$$

Consider the strain energy increase in the stiffness time variant as

(40) $$U\cdot t=\int_{0}^{\delta_{1}}Pd\delta \cdot t=\int_{0}^{\delta_{1}}K(t)\delta (t)d\delta \cdot t$$

So, it can be obtained

(41) $$\int_{0}^{\delta_{1}}K_{eq}\delta d\delta \cdot t=\int_{0}^{\delta_{1}}K(t)\delta (t)d\delta \cdot t$$

And since K(t) is a periodic function, according to Figure 12, Figure 13, Figure 14 and Figure 15, we see that K(t) is a trigonometric-like function, and its integral over one period is $\frac{K_{max}+K_{min}}{2}\cdot T$, so it can be further obtained

(42) $$K_{eq}=\frac{K_{\max}+K_{\min}}{2}$$

The further introduction of the coefficient κ, which makes $K_{min}=\kappa K_{max}$, can be further obtained:

(43) $$K_{eq}=\frac{\left(1+\kappa \right)}{2}K_{\max}$$

And the coefficient κ is mainly related to each parameter of the elastic ring, while the effect of each parameter is independent, so

(44) $$\kappa =\kappa_{1}\cdot \kappa_{2}\cdot \kappa_{3}\cdot \ldots \cdot \kappa_{n}$$

**Figure 17:**
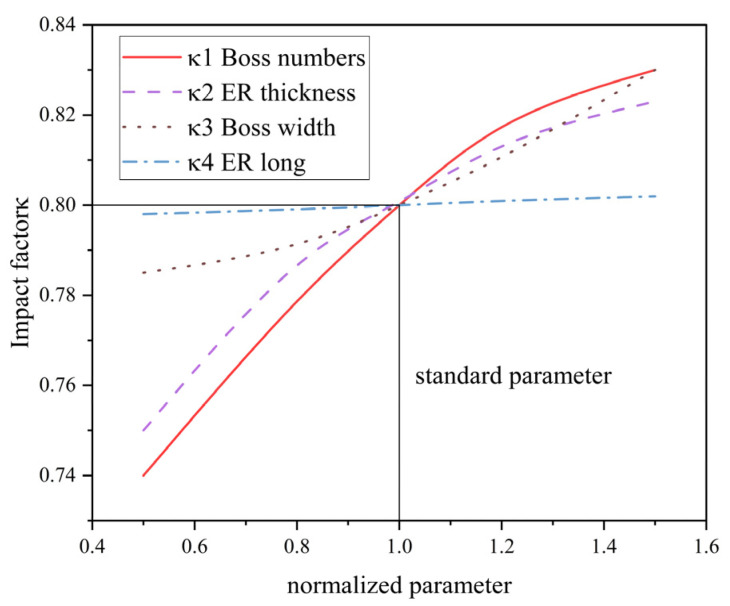
Influence factors corresponding to different parameters.

$\kappa_{i}\left(i=1,\;2,\ldots \;,n\right)$ indicates that the independent influence factor of each parameter, according to Figure 12, Figure 13, Figure 14, Figure 15 and Figure 16, can be obtained from the influence factor of the number of tabs, width, length, and the thickness of the elastic ring $\kappa_{1}$, $\kappa_{2},\;\kappa_{3},and\;\kappa_{4}$, as shown in Figure 17. The number of tabs being 8, the width being 10°, the thickness being 10 mm, and the length being 15 mm are used as the parameter normalization criteria, and their corresponding influence factor is 0.8.

**Figure 18:**
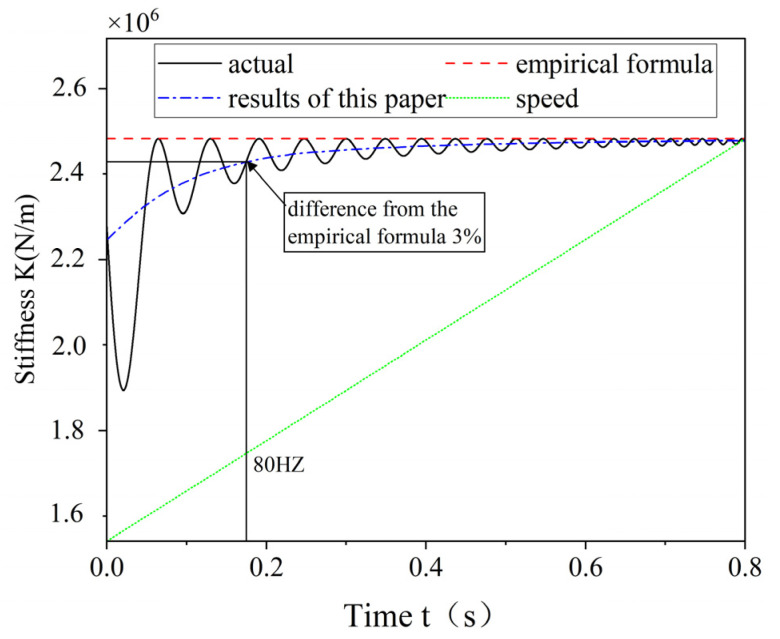
Comparison of equivalent methods.

When in the ramp-up phase, the frequency of K(t) also changes continuously, so the integral of the actual stiffness in one cycle also changes, and the time-varying stiffness characteristic of ERSFD is also weakened. The traditional method basically uses the maximum stiffness of the elastic ring. The trend of the stiffness equivalence method proposed in this paper and the traditional equivalence method when the rotor is in the speed-up phase is shown in Figure 18.

## 4. ERSFD Internal and External Oil Film Dynamics Characterization

### 4.1. Oil Film Pressure Distribution Inside and Outside the ERSFD

According to Equations (16) and (18), to obtain the stiffness and damping of the inner and outer oil films, the pressure of the inner and outer oil films must be obtained first, so the pressure distribution of the inner and outer oil films is analyzed first, and the deformation of the elastic ring is combined with the study of the generation and distribution of the oil film pressure.

**Figure 19:**
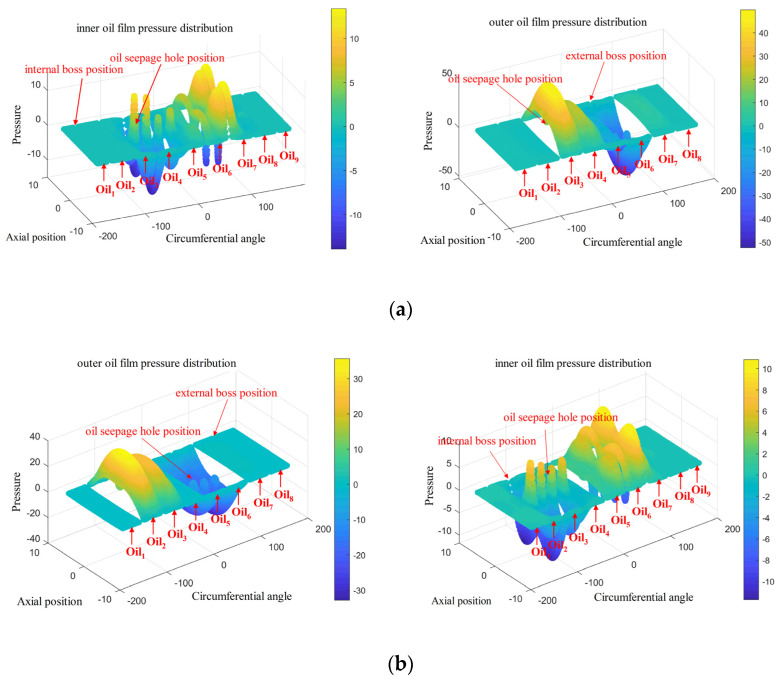
Pressure distribution of the oil film inside and outside the elastic ring. (**a**) Force on double tabs. (**b**) Force on a single tab.

Figure 19 shows the oil film pressure distribution of the general and special positions of the elastic ring force. The existence of the inner and outer tabs in the structure of the elastic ring divides the inner and outer oil films into eight chambers each, which are named ${Oil}_{i}(i=1,\;2,\;3,\ldots ,8)$ in turn. The oil film pressure is discontinuous in the whole circumferential direction due to the absence of oil film at the positions corresponding to the inner and outer tabs or the existence of an oil film with small thickness, which is generally ignored.

We can see from Figure 19 that at every moment, the outer oil film will have one oil chamber with the maximum pressure, such as ${Oil}_{3}$ in Figure 19a and ${Oil}_{2}$ in Figure 19b. And the pressure peak of the inner oil film will appear in the adjacent two oil cavities, such as ${Oil}_{6}$ and ${Oil}_{7}$ in Figure 19a,b, which indicates that the circumferential distribution pressure of the outer oil film always appears in the center of the oil cavity, while the maximum pressure position of the inner oil film appears in the adjacent area of the two oil cavities, that is, around the inner camber.

Different from the deformation of the elastic ring, the oil film not only changes in the circumferential direction but also in the axial direction, the oil film pressure is the largest in the position of the center, and the pressure decreases along the axial direction to the two sides, which is due to the existence of the oil seepage holes in the center position, and the slippery oil between each chamber can only flow through the seepage holes, so that the dynamic pressure around the seepage holes is the largest due to the flow of the slippery oil. However, the variation in oil film pressure occurs at the location of the oil weep hole. Due to the existence of the oil weep hole, the pressures of the inner and outer oil films at the oil weep hole will be kept the same. Take Figure 19a as an example, the chambers of the outer oil film ${Oil}_{3},\;{Oil}_{4}$ and the inner oil film ${Oil}_{3},\;{Oil}_{4}$ are connected, and it can be found that the chamber of the outer oil film ${Oil}_{3}$ shows a very significant decrease in oil film pressure at the location of the oil weep hole, whereas the chamber of the inner oil film ${Oil}_{3}$ shows a very significant increase in oil film pressure at the location of the oil weep hole.

**Figure 20:**
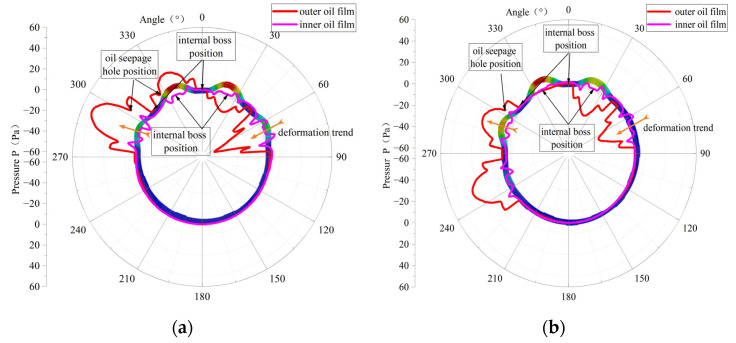
Oil film pressure distribution. (**a**) Force on double tabs. (**b**) Force on a single tab.

Figure 20 shows the distribution of the oil film pressure in the circumferential direction; the oil film pressure is mainly generated at the position of the outer ring of the elastic ring, while the oil film pressure in the inner ring is more often generated due to the passive linkage of the oil seepage holes.

From the figure, it can be seen that the maximum value of the external oil film pressure in the single-bump stress condition in the elastic ring deformation occurs in the largest adjacent oil cavity, with a maximum value of 50 Kpa; the oil film pressure distribution is not continuous in the deformation of the largest position in the emergence of the oil film pressure of the close to zero oil cavity.

In the case of double-bump force, the maximum value of oil film pressure appears in the adjacent oil cavity at the maximum deformation of the elastic ring, with a maximum value of 40 KPa, and its pressure distribution is more continuous.

In Figure 20, combined with the deformation condition of the elastic ring, it can be found that the generation of oil film pressure is always related to the deformation trend of the elastic ring, that is, related to the change in oil film thickness. For the outer oil film, since the deformation of the elastic ring will directly lead to a change in the oil film thickness, a larger oil film pressure will be generated.

As for the inner oil film, due to not only the deformation of the elastic ring but also the journal of the progress, the thickness of the oil film changes are very small, and due to the existence of oil seepage holes, when the pressure of the outer oil film increases, it will always be discharged through the oil seepage holes to the inner membrane of a certain amount of slippery oil to inhibit the inner layer of the oil film pressure generation.

**Figure 21:**
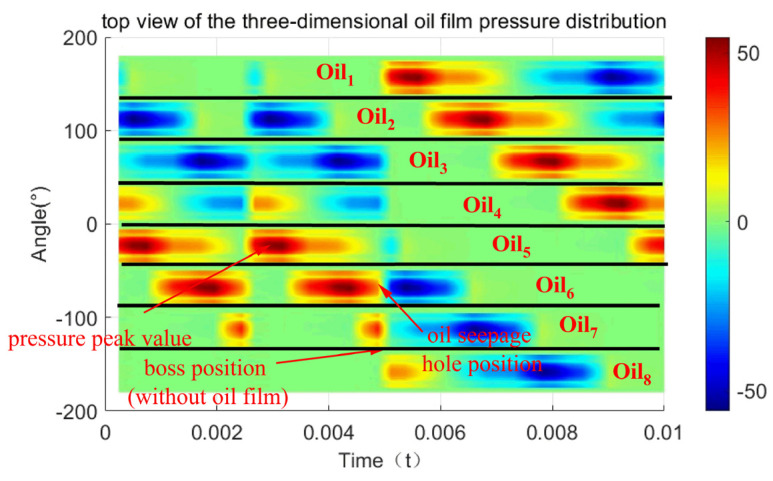
Oil film pressure variation in each chamber.

Figure 21 shows the variation in oil film pressure with time in the different chambers of the outer oil film.

It can be found that each oil chamber has a similar law of variation with time and a similar period, except that the phase at which the maximum value of the oil film pressure occurs varies, and the phase difference between the two neighboring oil chambers is just the angle corresponding to the center position of the two tabs.

The location of the film pressure maximum alternates from chamber to chamber, initially at ${Oil}_{5}$, but then at ${Oil}_{6}$, and so on in adjacent chambers. However, the maximum value of the oil film pressure remains constant, and the oil film pressure varies cyclically from chamber to chamber.

### 4.2. Time-Varying Characterization of Oil Film Stiffness and Damping

According to the above analysis, the pressure of the oil film is time varying, so the oil film stiffness and damping are analyzed next under the consideration of time varying.

**Figure 22:**
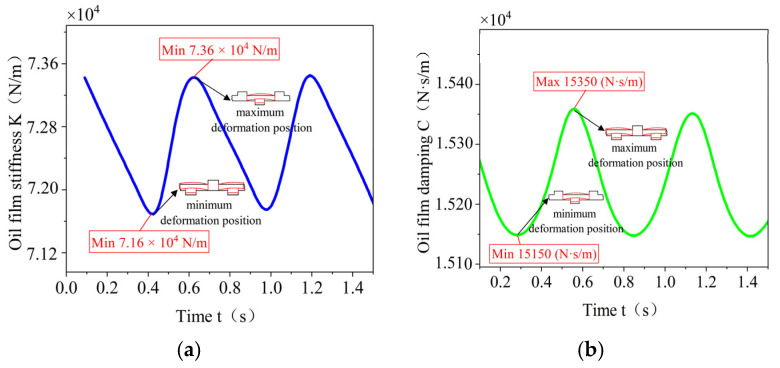
Oil film stiffness and damping. (**a**) Time-varying oil film stiffness curves. (**b**) Time-varying oil film damping curve.

As shown in Figure 22, the time-varying stiffness and time-varying damping curves of the inner and outer oil films are shown in Figure 22. The trends of the maximum stiffness and maximum damping of the oil film are very consistent, and the maximum value of the oil film stiffness and the maximum value of the damping both appear when the deformation of the elastic ring is the largest, and, at this time, its stiffness is $7.36\times 10^{4}\;N/m$, and the damping is 1535 N⋅s/m. The minimum values of oil film stiffness and damping both appear when the deformation of the elastic ring is the smallest, and then the stiffness is $7.16\times 10^{4}\;N/m,$ and the damping is 1515 N⋅s/m. Combined with the deformation of the elastic ring, it can be seen that the trends of the oil film stiffness and damping are opposite to the time-varying trend of the elastic ring stiffness.

Combined with Figure 22, although the oil film stiffness and damping are also time varying, the time-varying characteristics of the oil film stiffness and damping are very weak, the difference between the maximum and minimum values of the oil film stiffness is only 3% under the most basic elasticity ring parameter, and the amount of variation in the oil film damping is lower than 2%. The equivalence method given in the previous section is directly adopted in the case of the further discussion of the influence of different elasticity ring parameters on the oil film stiffness and damping, and a coefficient of 0.97 is selected (κ=0.97) for the time-varying equivalence of oil film stiffness and damping.

### 4.3. Influence of the Structural Parameters of the Elastic Ring on the Damping and Stiffness of the Inner and Outer Oil Films

The effects of varying the structural parameters of different elastic rings on the damping and stiffness of the inner and outer oil films are further investigated.

**Figure 23:**
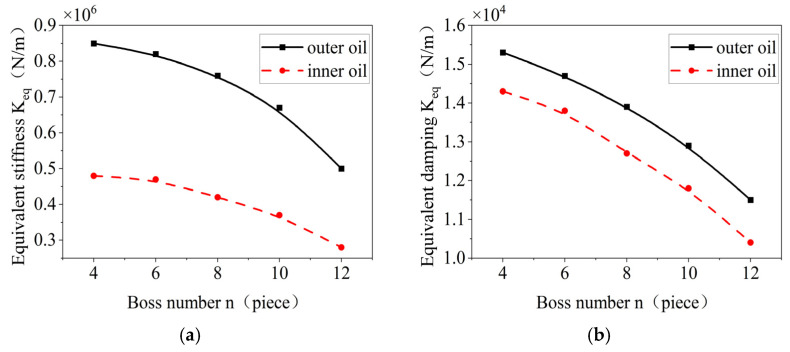
Effect of the number of bosses on the oil film stiffness and damping inside and outside. (**a**) Changes in equivalent stiffness. (**b**) Time-varying oil film damping curve.

The effects of the number of bosses on the stiffness and damping of the inner and outer oil films of the elastic ring are shown in Figure 23. With the increase in the number of bosses, the inner and outer equivalent oil film and the stiffness of the elastic ring tend to decrease due to the fact that when the number of bosses is increased, it results in the reduction in the volume of the oil film chamber, which results in the reduction in the pressure that can be supplied by the oil film. Also, the overall deformation of the elastic ring decreases due to the increase in the number of tabs.

**Figure 24:**
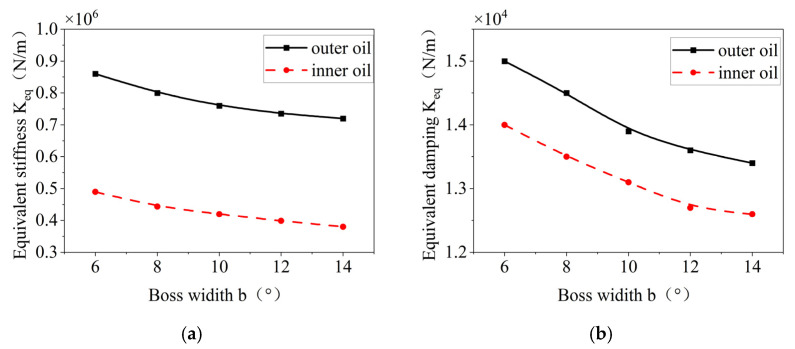
Effect of boss width on the oil film stiffness and damping inside and outside. (**a**) Changes in equivalent stiffness. (**b**) Variation in equivalent damping.

The effects of the tab width of the elastic ring on the stiffness and damping of the inner and outer oil films are shown in Figure 24; and an increase in the tab width leads to a decrease in the equivalent oil film and stiffness, which is also affected by the same factor as the increase in the number of tabs, and when the oil film width is increased, the oil film area decreases, and the deformation of the elastic ring is weakened.

**Figure 25:**
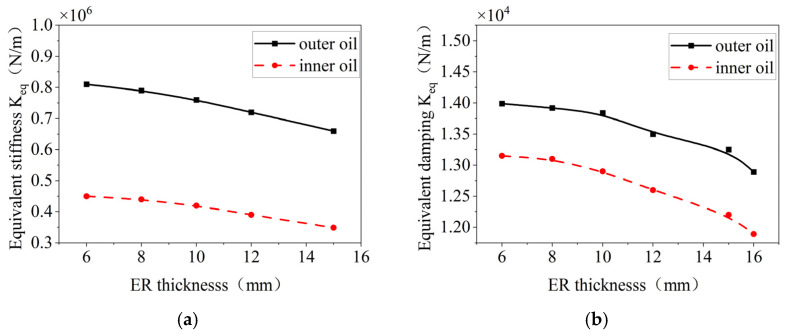
Effect of boss width on the oil film stiffness and damping inside and outside. (**a**) Change in equivalent stiffness. (**b**) Variation in equivalent damping.

The effects of the thickness of the elastic ring on the stiffness and damping of the inner and outer oil films are shown in Figure 25, where a decrease in the equivalent stiffness and damping of the oil film occurs as the thickness increases.

**Figure 26:**
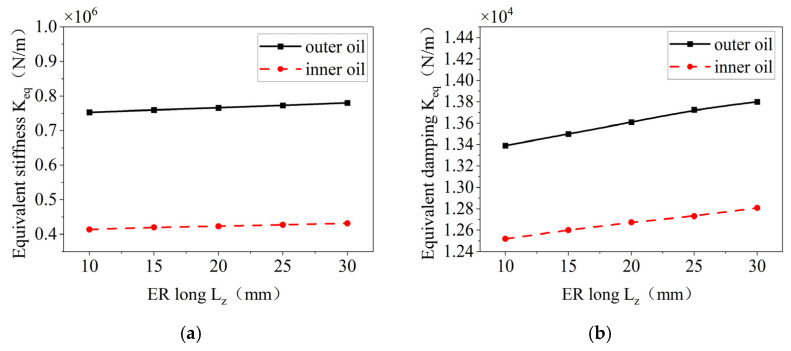
Effect of elastic ring length on stiffness and damping of inner and outer oil films. (**a**) Change in equivalent stiffness. (**b**) Change in damping.

The effects of the axial length of the elastic ring on the stiffness and damping of the inner and outer oil films are shown in Figure 26. With an increase in the axial length, the equivalent stiffness and damping of the oil film appear to increase to some extent, but the change is not very obvious. This is due to the fact that the increase in axial length leads to an increase in the region of the oil film, but the deformation of the elastic ring does not change much. Therefore, the change in the equivalent damping and stiffness of the oil film is not very obvious.

According to Equations (16) and (18), in addition to the structural parameters of the elastic ring such as the number of tabs, the thickness of the elastic ring, and so on, the eccentricity of the oil film also has a very significant effect on the damping and stiffness of the inner and outer oil films. Therefore, on the basis of studying the structural parameters of the elastic ring, the influence of the eccentricity of the oil film on the characteristics of the inner and outer oil films is additionally investigated.

**Figure 27:**
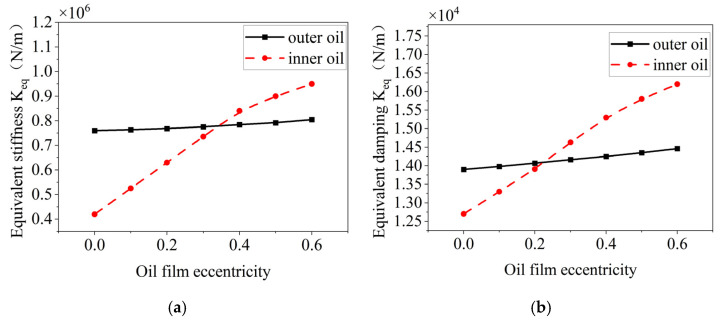
Effect of oil film eccentricity on the oil film stiffness and damping inside and outside. (**a**) Trends in equivalent stiffness. (**b**) Trend in equivalent damping.

The effect of the oil film eccentricity on the stiffness and damping of the inner and outer oil films is shown in Figure 27, which shows that as the eccentricity of the oil film increases, the equivalent stiffness and damping of the inner and outer oil films are increased to some extent. The change in the equivalent stiffness and damping of the inner oil film is more obvious, which is due to the fact that the change in the eccentricity of the oil film will directly lead to a change in the oil film thickness of the inner oil film.

In summary, the trends and sensitivities of the relevant parameters of the elastic ring and the oil film on the stiffness and damping of the oil film are inconsistent, in which the increase in the number of elastic ring bosses, the width of the bosses, and the thickness of the elastic ring leads to a decrease in the equivalent damping and stiffness of the oil film, with the number of bosses having the greatest influence and the thickness of the elastic ring having the weakest influence. The increase in the length of the elastic ring and the eccentricity of the oil film will lead to an increase in the equivalent stiffness and damping of the oil film, and the eccentricity of the oil film has a very significant effect on the equivalent stiffness and damping of the inner oil film.

## 5. Conclusions

This paper mainly focuses on the ERSFD structure in aero-engine combined support, analyzes the different force states of the elastic ring from the two-way fluid–solid coupling of ERSFD, finds the time-varying stiffness of the elastic ring, and gives the stiffness equivalent method of the time-varying stiffness, and based on this, it analyzes the influence of the structural parameters of the elastic ring on the stiffness of the elastic ring. The pressure control equations of the inner and outer oil films, considering the deformation of the elastic ring, are established, and the pressure distribution and generation law of the inner and outer oil films as well as the influence of the structural parameters on the stiffness and damping of the inner and outer oil films are investigated. The main conclusions are as follows:

(1) During operation, the elastic ring experiences different contact states, classified as single-tab and double-tab contact. The difference between the maximum and minimum stiffness in these two states can be as much as 10%.
(2) The elastic ring exhibits time-varying stiffness characteristics. Based on this, a new equivalent stiffness method is proposed that takes time variation into account. This method allows the equivalent stiffness to be calculated based on the ring’s structural parameters and is over 5% more accurate than traditional methods at low speeds.
(3) The number of inner and outer tabs, the thickness of the elastic ring, and the width of the tabs all have a significant effect on the deformation of the ring. However, only the number of tabs and the ring thickness have a noticeable impact on its time-varying stiffness. These two parameters should be given special attention during the design process.
(4) Increasing the number of tabs, the ring thickness, or the width of the inner and outer tabs results in a decrease in both the pressure and damping of the inner and outer oil films. These trends are consistent for both films. Conversely, increasing the axial length of the ring or the oil film eccentricity leads to higher stiffness and damping in both films.
(5) The pressure in the outer oil film is primarily influenced by the elastic ring’s deformation and the size of the oil cavity, while the inner oil film pressure is most sensitive to oil film eccentricity. In design, increasing oil film eccentricity can help enhance overall stiffness and damping. However, excessive eccentricity may introduce nonlinear effects.

This paper provides a theoretical basis for the accurate calculation of the dynamic characteristics of ERSFD combined support systems for aero-engines.

## Figures and Tables

**Table 1:** Variation in deformation and stiffness of the elastic ring under different parameters.

Variable	Parameters	Deformation (mm)	Degree (%)	$\mathrm{Rigidity}\;10^{5}\;N/m$	Degree (%)	Variable	Parameters	Deformation (mm)	Degree (%)	Rigidity $10^{5}\;N/m$	Degree (%)
n	4	0.0343	281	0.812	−65	b	06	0.0112	24	1.880	−17
	6	0.0168	86	1.333	−42		08	0.0101	12	2.076	−8
	8	0.0090	0	2.267	0		10	0.0090	0	2.267	0
	12	0.0017	−81	12.050	430		12	0.0076	−15	2.801	24
$L_{z}$	10	0.0094	4	2.156	−5	s	08	0.0254	180	0.840	−62
	15	0.0090	0	2.267	0		10	0.0179	98	1.190	−47
	20	0.0085	5	2.591	14		12	0.0132	47	1.610	−30
	25	0.0081	10	2.720	20		15	0.0090	0	2.267	0

## Data Availability

The original contributions presented in this study are included in the article. Further inquiries can be directed to the corresponding author.
